# Association between single-nucleotide polymorphisms and early spontaneous hepatitis B virus e antigen seroconversion in children

**DOI:** 10.1186/1756-0500-7-789

**Published:** 2014-11-06

**Authors:** Haruki Komatsu, Jun Murakami, Ayano Inui, Tomoyuki Tsunoda, Tsuyoshi Sogo, Tomoo Fujisawa

**Affiliations:** Department of Pediatrics, Toho University, Sakura Medical Center, 564-1 Shimoshizu Sakura, Chiba, 285-8741 Japan; Division of Hepatology and Gastroenterology, Department of Pediatrics, Eastern Yokohama Hospital, Yokohama, Japan; Division of Pediatrics and Perinatology, Faculty of Medicine, Tottori University, Yonago, Japan

**Keywords:** Hepatitis B virus, Single-nucleotide polymorphism, Human leukocyte antigen, IL28B, IL10, HBeAg, Seroconversion, Children, HLA-DP

## Abstract

**Background:**

The disease progression following hepatitis B virus (HBV) infection is associated with single-nucleotide polymorphisms (SNPs). However, the role of SNPs in chronic HBV infection in children remains unclear. Here, we investigate the association between SNPs and early spontaneous hepatitis B e antigen (HBeAg) seroconversion in children with chronic hepatitis B infection.

**Methods:**

This was a retrospective cohort study. We genotyped seven SNPs in the following genes, interleukin (*IL*)-*10* (rs1800871 and rs1800872), human leukocyte antigen (*HLA*)-*DPA1* (rs3077), *HLA-DPB1* (rs9277535), *HLA*-*DQB2* (rs7453920), *HLA-DQB1* (rs2856718), and *IL28B* (rs8099917), in patients with chronic HBV infection using PCR and sequencing. These variants were analyzed for an association with early HBeAg seroconversion in children.

**Results:**

Of 225 Japanese patients with chronic hepatitis B virus infection (male/female: 105/120, median age at initial visit: 6 years; range 0–44 years), 52 achieved spontaneous HBeAg seroconversion at the age of 10 years or younger (G1: early seroconversion group), and 57 did not achieve spontaneous HBeAg seroconversion under the age of 20 years (G2: late or no seroconversion group). Of the seven SNPs, only the *HLA-DPA1* SNP displayed a low p-value (P = 0.070), but not significant, to have early HBeAg seroconversion in the dominant model and in the allele model (P =  0.073) using the chi-square test. The association study found a low p-value, but not significant, to have early HBeAg seroconversion in the dominant model for *HLA-DPA1* (genotype TC + TT vs. CC, P = 0.070, odds ratio: 2.016, 95% confidence interval: 0.940-4.323) using a logistic regression model.

**Conclusion:**

Although the *HLA-DPA1* SNP did not show a statistically significant association with early HBeAg seroconversion in this study, the *HLA-DPA1* SNP might increase the likelihood of achieving early spontaneous HBeAg seroconversion in children.

## Background

Although universal vaccination with the hepatitis B vaccine has been introduced in almost all countries, more than 260 million people are suffering from chronic hepatitis B virus (HBV) infection worldwide. Adults with chronic HBV infection have a 15 to 20% risk of dying from HBV-related liver disease such as liver cirrhosis or hepatocellular carcinoma (HCC) [1]. In contrast, during childhood and adolescence, 3 to 5% and 0.01 to 0.03% of patients with chronic HBV infection develop cirrhosis and HCC, respectively [2]. Although host factors (e.g., male sex, older age, Asian or African ancestry, and family history of HCC) and viral factors (e.g., higher viral load, HBV genotype, longer duration of infection, and co-infection with hepatitis C virus, human immunodeficiency virus, or hepatitis D virus) are known risk factors for HCC [3], predicting which chronic HBV-infected children should be treated is difficult. The European guidelines for children with chronic HBV infection recommend basing the decision to start treatment on ALT levels, HBeAg positivity, HBV-DNA levels, liver histology, family history of HCC, co-existing liver disease, and the patient’s treatment history [2]. In particular, HBeAg-seroconversion is usually accompanied by the remission of liver disease and confers a favorable outcome in children as well as adults [4].

Single nucleotide polymorphisms (SNPs) are well known to affect disease progression following HBV infection. The cytokine production-induced cell-mediated antiviral immune response plays a crucial role in the control of viral infection. Genetic variants of cytokines such as interleukin (IL)-10 and tumor necrosis factor-α gene have been associated with the outcome of HBV infection [5–11]. Moreover, recent genome-wide association studies showed that genetic variants of human leukocyte antigen (*HLA*)-*DP* and *HLA-DQ* are strongly associated with the outcome of HBV infection in adults from Japan, Korea, and China [12–21]. However, whether *HLA*-*DP* and *HLA-DQ* genetic variants influence the outcome of HBV infection in children remains unknown. In this study, we retrospectively evaluated the effect of genetic variants of *IL-10*, *HLA-DP, HLA-DQ,* and *IL-28B* on early spontaneous hepatitis B e antigen (HBeAg) seroconversion in children with chronic HBV infection.

## Methods

### Patients

The present study was a retrospective study. The diagnosis of chronic HBV infection was based on the detection of serum hepatitis B surface antigen on 2 occasions at least 6 months apart. Moreover, the patients were regularly followed for the measurement of serum ALT levels, HBeAg, anti-HBe antibodies, and AFP levels every 6 months. Patients with a history of antiviral treatment for hepatitis B infection were excluded. All patients were negative for anti-HCV antibodies. Patients with autoimmune hepatitis, Wilson’s disease, and primary sclerosing cholangitis were excluded. The co-infection of HBV and hepatitis D virus (HDV) is extremely rare in Japan. Therefore, the co-infection of HBV and HDV was not excluded. The study protocols were approved by the ethical committee of Eastern Yokohama Hospital (No. 2011025) and Tottori University (G141), and performed in accordance with the ethical guidelines of the 1975 Declaration of Helsinki. Written informed consent was obtained from all parents or legal guardians of children. In addition, we obtained written informed consent from each adult patient participating in this study.

### DNA extraction and SNPs analysis

Whole blood or serum was used for DNA extraction. Briefly, genomic DNA was extracted from 1 mL of peripheral whole blood using a Puregene blood core kit (Qiagen, Hilden, Germany) or from 200 μL of serum using a QIAamp DNA blood kit (Qiagen) according to the manufacturer’s instructions. The DNA was eluted in 200 μL of elution buffer. Seven SNPs, *IL-10* (IL-10-819: rs1800871), *IL-10* (IL-10-592: rs1800872), *HLA*-*DPA1* (rs3077), *HLA-DPB1* (rs9277535), *HLA*-*DQB2* (rs7453920), *HLA-DQB1* (rs2856718), and *IL-28B* (rs8099917), were assessed in this study.

PCR was performed using a 50 μL reaction mixture containing 25 μL of AmpliTaq Gold 360 Master Mix (Applied Biosystems, Foster City, CA), 25 pmol of each primer, and 5 μL of extracted DNA. When whole blood samples were used for PCR, a single-round PCR was performed. When serum samples were used for PCR, nested PCR was performed. The PCR amplification was performed using the following protocol: an initial pre-cycle incubation of 95°C for 10 min, followed by 40 cycles of denaturation at 95°C for 30 s, annealing at 55°C for 30 s, and extension at 72°C for 60 s. Then, 1 μL of the first PCR reaction product was reamplified with the inner primers for 40 cycles under the same reaction conditions used in the first-round PCR. The amplified PCR products were purified using a QIAquick gel extraction kit (QIAGEN) and then used for direct sequencing in the forward and reverse directions. Nested primer sets were synthesized to amplify each region. An internal primer-pair was used for the single-round PCR amplification of DNA extracted from whole blood. The sequences of the PCR primers are shown in Table 1. The levels of serum HBV DNA was measured by COBAS TaqMan HBV DNA test (detection range from 2.1 to 9.0 log copies/mL).

**Table 1:** **Primers for amplification and sequencing**

SNP ID			sequence (5 'to 3')
*IL-10-819* (rs1800871)	External	forward	TCAACTTCTTCCACCCCATC
		reverse	GGCACATGTTTCCACCTCTT
	Internal	forward	GGGTGAGGAAACCAAATTCTC
		reverse	TGCACTTGCTGAAAGCTTCTT
*IL-10-592* (rs1800872)	External	forward	TGGAAACATGTGCCTGAGAA
		reverse	CAGTGACGTGGACAAATTGC
	Internal	forward	AAAGGAGCCTGGAACACATC
		reverse	CCTTAGGTCTCTGGGCCTTA
*HLA-DPA1* (rs3077)	External	forward	CTGAACTCCAGCTGCCCTAC
		reverse	CTCCCCGCTCTGAAATACTG
	Internal	forward	AACTCCAGCTGCCCTACAAA
		reverse	GGATAAAAGGCTCAATGAAAGG
*HLA-DPB1* (rs9277535)	External	forward	GGGCCTGTTACACATGACACT
		reverse	TGGATGCATTCAAAAGTCCA
	Internal	forward	TGCCCCCAAATCAAGTTTAG
		reverse	TGGCACACAAAGAAAATGGT
*HLA-DQB2* (rs7453920)	External	forward	ACGCGAAATTGAGTTCTTGG
		reverse	CAGGCATGGGTTTACTTGGT
	Internal	forward	GGTAAGAGGGAAAGCCCAGT
		reverse	CTGTCTCCGAGATTCCCAAG
*HLA-DQB1* (rs2856718)	External	forward	TTGGCCAGAGTATGCTTTCA
		reverse	TTTGCCCTGAGGTCTATGCT
	Internal	forward	TATGCTTTCACCAACTTCCTTCAC
		reverse	GAGCTCCCTCTGGCAGGTT
*IL-28B* (rs8099917)	External	forward	GTGCATATGTTTTCTGAC
		reverse	GAGGCCCCTCACCCATGC
	Internal	forward	AAGTAACACTTGTTCCTTGTAAAAGATTCC
		reverse	CGCTATAATTAAAGATGTGGGAGAATGCAA

### Statistical analysis

To characterize the patients’ background, statistical analyses were performed with StatMate IV for Windows (Advanced Technology for Medicine & Science, Tokyo, Japan), Microsoft Office Excel 2007, and STATA/MP software (version 13.1; StataCorp, College Station, TX). Categorical variables and non-categorical variables were compared between groups using the Yates corrected chi-square test and Mann–Whitney U test, respectively. The multivariate test is by maximum likelihood logistic regression (association of all of the factors with “Early” versus “Late or no” group). Moreover, all statistical analyses for SNPs were performed using SNPAlyze Ver.8 (DAYNACOM, Chiba, Japan). We tested the genotypic distributions for Hardy-Weinberg equilibrium (HWE) using the Yates corrected chi-square test. The difference between the case–control groups in terms of the distribution of genotypes was analyzed using the Cochran-Armitage trend test if HWE did not hold in the combined case–control population [22]. Logistic regression was performed for the comparison between the case group (G1: early seroconversion) and the control group (G2: late or no seroconversion). A p-value of 0.05 or less was considered statistically significant. The sample size estimation was calculated using G*Power 3.1.9.2 (http://www.gpower.hhu.de/) [23].

## Results

### Patient characteristics

**Figure 1:**
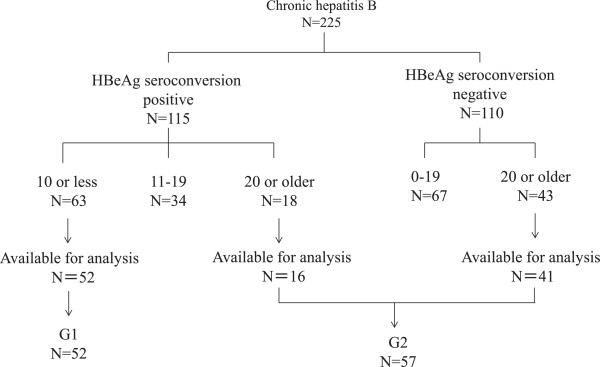
**Classification of the subjects based on hepatitis B e antigen seroconversion.**

Between 1981 and 2012, a total of 225 Japanese patients comprising children, adolescents, and mothers (male/female: 105/120, age: 0–44 years, median age: 6 years, follow-up period: 1–25 years, median: 8 years) with chronic HBV infection were followed in the Pediatric Departments of Eastern Yokohama Hospital and Tottori University Hospital. Of the 225 patients with chronic HBV infection, 115 achieved HBeAg seroconversion and 110 did not achieve HBeAg seroconversion during the follow-up period. Of the 115 patients with HBeAg seroconversion, 63 achieved HBeAg seroconversion at the age of 10 years or younger, 34 achieved HBeAg seroconversion between the ages of 11 and 19 years, and 18 achieved HBeAg seroconversion at the age of 20 years or older. Of the 110 patients without HBeAg seroconversion during the follow-up period, 67 were under the age of 20 years, and 43 were 20 years or older. Of the 63 patients who achieved HBeAg seroconversion at the age of 10 years or younger, 52 were available for evaluation and classified into the early seroconversion group (G1). Of the 18 patients who achieved HBeAg seroconversion at the age of 20 years or older, 16 were available for evaluation. Of the 43 patients without HBeAg seroconversion who were 20 years or older, 41 were available for evaluation. A total of 57 patients were classified into the late or no seroconversion group (G2). The classification scheme of the patients based on HBeAg seroconversion is shown in Figure 1.

The patients characteristics in individual groups were shown in Table 2. In G1, the sources of HBV infection were mother-to-child transmission (n = 43), family contact (n = 5), unknown (n =3), and blood transfusion (n = 1). In G2, the sources were mother-to-child transmission (n = 39), unknown (n = 14), and family contact (n = 4). Of the 57 patients belonging to G2, 37 were HBV carrier mothers. Therefore, there was a significant difference in the sex ratio between G1 and G2. The levels of ALT at the initial visit were significantly higher in the G1 group than the G2 group. There was no significant difference in the duration of the follow-up period or HBV genotype between the G1 and G2 groups. At the last visit, 23 of 57 patients in the G1 were positive for serum HBV DNA and all of G2 patients were positive for serum HBV DNA. The levels of serum HBV DNA were 4.8 (median) log copies/ml and 8.5 (median) log copies/ml in the G1 and G2, respectively. There was a statistical significance in age at initial visit, gender, ALT level at initial age, serum HBV DNA level at last visit, and mother-to-child transmission between G1 and G2 in univariate analysis . Multivariate analysis showed that a statistical significance between G1 and G2 was detected in gender, duration of the follow-up period, serum HBV DNA level, and mother –to-child transmission.

**Table 2:** **Patients characteristics in individual groups**

		G1: Early (N = 52)	G2: Late or no seroconversion (N = 57)	Univariate	Multivariate
				P value	P value
Age at initial visit (year)		0-10 (Median 2.8)	0-44 (Median 28)	< 0.00005	****
Gender (Male/Female)		28/24	5/52	< 0.00005	0.001
ALT level (IU/L) at initial visit		10-725 (Median 80.5)	7-767 (Median 23)	< 0.00005	0.489
Duration of follow-up period (year)		1-21 (Median 10.0)*	1-25 (Median, 6.0)	0.6218	0.013
Age at HBe seroconversion		1-10 (Median 6.5)	21-34 (Median 25)**	Not done	Not done
Serum HBV DNA level at last visit (log copies/mL)		2.5-8.7 (Median 4.8)***	3.0-9.0 (Median 8.5)	< 0.00005	0.003
Transmission route	Mother-to-child	43	37	Mother-to-child	
	Household contacts	5	4	v.s. Non Mother-to-child	
	Blood transfusion	1	0	0.036	0.016
	Unknown	3	16		
HBV Genotype	A	0	1	Genotype C	
	B	7	10	v.s. Non genotype C	
	C	44	45	0.445	0.221
	D	1	1		

*Data of 51 patients were available.

**Of the 57 patients, 17 patients archived seroconversion.

*** Of the 57 patients, 23 patients were positive for serum HBV DNA and the remaining 28 patients were negative for HBV DNA.

****Omitted from model in order to permit convergence to finite maximim likelihood estimates.

### Hardy-Weinberg equilibrium

The HWE proportions are shown in Table 3. Of the 7 SNPs, 5 (*IL-10-819*: rs1800871, *HLA-DPA1*: rs3077, *HLA-DPB1*: rs9277535, *HLA-DQB1*: rs2856718, and *IL28B*: rs8099917) had genotype distributions that did not significantly deviate from HWE (*p* >0.05) in either G1 or G2. However, the remaining 2 SNPs showed a significant deviation in G1 and/or G2. These findings suggest that a chi-square test was inappropriate for the analysis of the case–control association for *IL10-592* (rs1800872) and *HLA*-*DQB2* (rs7453920).

**Table 3:** **The results of the Hardy-Weinberg equilibrium proportions**

SNP ID	Genotype	No. of G1 early seroconversion genotype frequency (%)	P value	No. of G2 late or no seroconversion genotype frequency (%)	P value
IL-10-819	TT	24 (46.2)		25 (43.9)	
(rs1800871)	CT	18 (34.6)	0.126	22 (38.6)	0.3116
	CC	10 (19.2)		10 (17.5)	
IL-10-592	AA	23 (44.2)		26 (45.6)	
(rs1800872)	CA	14 (26.9)	0.003	21 (36.8)	0.221
	CC	15 (28.8)		10 (17.5)	
HLA-DPA1	CC	22 (42.3)		34 (59.6)	
(rs3077)	TC	27 (51.9)	0.267	22 (38.6)	0.424
	TT	3 (5.8)		1 (1.8)	
HLA-DPB1	GG	27 (51.9)		26 (45.6)	
(rs9277535)	AG	17 (32.7)	0.147	21 (36.8)	0.221
	AA	8 (15.4)		10 (17.5)	
HLA-DQB2	GG	43 (82.7)		50 (87.7)	
(rs7453920)	GA	0	1.736 × 10^−11^	1 (1.8)	4.409 × 10-^10^
	AA	9 (17.3)		6 (10.5)	
HLA-DQB1	AA	18 (34.6)		18 (31.6)	
(rs2856718)	GA	24 (46.2)	0.913	30 (52.6)	0.742
	GG	10 (19.2)		9 (15.8)	
IL-28B	TT	39 (75.0)		44 (77.2)	
(rs8099917)	GT	10 (19.2)	0.176	13 (22.8)	0.752
	GG	3 (5.8)		0	

### Association between SNPs and HBeAg seroconversion

None of the five SNPs that conformed to HWE expectations (*IL-10-819*: rs1800871, *HLA-DPA1*: rs3077, and *HLA-DPB1*: rs9277535, *HLA-DQB1*: rs2856718, and *IL28B*: rs8099917) had a significant association with early seroconversion in the dominant, recessive, allele, and genotype models using the Yates corrected chi-square test (Table 4). However, weak associations with early HBeAg seroconversion were observed in the dominant model (P = 0.070) and the allele model (P = 0.073) for the *HLA-DPA1* SNP. In addition, the AIC value (IM: independent model – DM: dependent model) was >0 in both models for the HLA-DPA1 SNP (Table 3). Because an AIC value >0 indicates that a genetic polymorphism and a disease are dependent [24], the *HLA-DPA1* SNP (rs3077) shows a tendency to have early spontaneous HBeAg seroconversion. The Cochran-Armitage trend test was used to evaluate the difference between the case and control groups (G1 vs. G2) in terms of the distribution of genotypes for the *IL10-592* SNP and *HLA*-*DQB2* SNP. However, neither of these SNPs showed a significant association (*IL10-592*; P = 0.405, *HLA*-*DQB2*: P = 0.374).

**Table 4:** **Association between SNPs and HBeAg seroconversion using Akaike’s information criteria and chi-square test**

SNP ID	Dominant model			Recessive model			Recessive model			Genotype model	
	AIC	Chi-squire test		AIC	Chi-squire test		AIC	Chi-squire test		AIC	Chi-squire test
	(IM-DM)	P value	OR (95% CI)	(IM-DM)	P value	OR (95% CI)	(IM-DM)	P value	OR (95% CI)	(IM-DM)	P value
IL-10-819	−1.942	0.810	0.912 (0.428-1.941)	−1.948	0.820	1.119 (0.424-2.953)	−1.998	0.963	0.987 (0.569-1.723)	−3.808	0.909
(rs1800871)											
HLA-DPA1	1.289	0.070	2.016 (0.940-4.325)	−0.716	0.266	3.429 (0.345-34.042)	1.216	0.073	1.743 (0.946-3.211)	−0.080	0.145
(rs3077)											
HLA-DPB1	−1.567	0.510	0.777 (0.366-1.650)	−1.908	0.762	0.855 (0.309-2.362)	−1.565	0.510	0.828 (0.471-1.453)	−3.566	0.805
(rs9277535)											
HLA-DQB1	−1.887	0.736	0.872 (0.392-1.938)	−1.777	0.636	1.270 (0.471-3.422)	−1.999	0.976	1.008 (0.589-1.727)	−3.509	0.782
(rs2856718)											
IL-28B	−1.928	0.788	1.128 (0.467-2.724)	2.534	0.066	ND	−1.253	0.387	1.413 (0.643-3.099)	0.623	0.176
(rs8099917)											

ND: no data.

A logistic regression model was also used to evaluate the association between SNPs and HBeAg seroconversion (Table 5). Although no statistically significant association between SNPs and early spontaneous HBeAg seroconversion was observed in the logistic regression model, the *HLA-DPA1* SNP (rs3077) showed a low p-value to have early spontaneous HBeAg seroconversion in the dominant model (genotype TC + TT vs. CC, P = 0.070, odds ratio: 2.016, 95% confidence interval: 0.940-4.323). Taken together, these findings suggest that the *HLA-DPA1* SNP (rs3077) has a possibility to induce early spontaneous HBeAg seroconversion in children.

**Table 5:** **Association between SNPs and HBeAg seroconversion using logistic regression model**

SNP (ID)	Dominant model			Recessive model			Genotype model		
		P value	OR (95% CI)		P value	OR (95% CI)		P value	OR (95% CI)
IL-10-819 (rs1800871)	CT + CC/TT	0.810	0.912 (0.428-1.941)	CC/TT + CT	0.820	1.120 (0.424-2.953)	CT/TT	0.909	0.852 (0.369-1.970)
							CC/TT		1.042 (0.368-2.948)
IL-10-592 (rs1800872)	CA + CC/AA	0.885	1.058 (0.497-2.252)	CC/AA + CA	0.160	1.905 (0.768-4.726)	CA/AA	0.306	0.754 (0.313-1.814)
							CC/AA		1.696 (0.638-4.504)
HLA-DPA1 (rs3077)	TC + TT/CC	0.070	2.016 (0.940-4.323)	TT/CC + TC	0.257	3.429 (0.3457-33.999)	TC/CC	0.141	1.897 (0.872-4.127)
							TT/CC		4.636 (0.454-47.397)
HLA-DPB1 (rs9277535)	AG + AA/GG	0.510	0.777 (0.366-1.650)	AA/GG + AG	0.761	0.855 (0.309-2.362)	AG/GG	0.805	0.780 (0.338-1.799)
							AA/GG		0.770 (0.263-2.256)
HLA-DQB2 (rs7453920)	GA + AA/GG	0.459	1.495 (0.514-4.352)	AA/GG + GA	0.304	1.779 (0.587-5.395)	GA/GG	ND	ND
							AA/GG		ND
HLA-DQB1 (rs2856718)	GA + GG/AA	0.736	0.872 (0.392-1938)	GG/AA + GA	0.636	1.270 (0.471-3.422)	GA/AA	0.782	0.80 (0.343-1.863)
							GG/AA		1.111 (0.365-3.380)
IL-28B (rs8099917)	GT + GG/TT	0.789	1.128 (0.467-2.724)	GG/TT + GT	ND	ND	GT/TT	ND	ND
							GG/TT		ND

ND: no data.

Because genotype GA of *HLA*-*DQB2* (rs7453920) in G1 and genotype GG of *IL28B* (rs8099917) in G2 were not detected, there are no data for the recessive model for *IL28B* in Table 4, the genotype model for *HLA*-*DQB2* in Table 5, or the recessive model and genotype model for *IL28B* in Table 4.

## Discussion

In this study, we evaluated retrospectively whether the genetic variants of *IL-10*, *HLA-DP*, *HLA-DQ*, and *IL-28B* could influence early spontaneous HBeAg seroconversion in children with chronic HBV infection. We thought that this SNP analysis needed 2 groups which had distinctly different phenotypes to archive a significant result. One is the early spontaneous HBeAg seroconversion group. The other is late or no HBeAg seroconversion group. Although there was no clinical significance of 10 and 20 of age, the patients with HBeAg seroconversion who are 10 or less of age were defined simply as “early HBeAg seroconversion” in order to obtain appropriate sample size of both groups. Initially, we compared the “early HBeAg-seroconversion group (G1: N = 52)” with the “no HBeAg-seroconversion group (N = 43)”. Although the odds ratio showed a high value for a few SNPs, the difference was not significant. We thought that the statistical power was insufficient due to the small sample size. Therefore, we added the patients with late HBeAg-seroconversion (N = 16) to the “no HBeAg-seroconversion group”. Accordingly, G2 (N = 57) was defined as the “late or no HBeAg-seroconversion group”. Although the *HLA-DPA1* (rs3077) SNP did not show a statistically significant association with early spontaneous HBeAg seroconversion, this study showed that the *HLA-DPA1* (rs3077) SNP displayed a low p-value to have early spontaneous HBeAg seroconversion in children. This finding is consistent with the results of previous studies that have reported that genetic variants of *HLA-DP* are strongly associated with the outcomes of HBV infection in Asian adult populations [12, 13, 18]. The genetic variants of *HLA-DP* loci contribute to the risk of persistent HBV infection. The T alleles of rs3077 (*HLA-DPA1*) and rs9277535 (*HLA-DPB1*) are associated with a decreased risk of chronic HBV infection [12, 13, 15, 17, 21, 25]. However, the assertion that these genetic variants are strongly associated with disease progression and HCC development in adults is controversial [16–18, 20]. Hu et al. showed that *HLA-DPA1* rs3077 is significantly associated with persistent HBV infection and HCC development [17]. In contrast, several studies have reported that there is no significant association between *HLA-DP* variants and HCC development [16, 18, 20]. The association between *HLA-DP* variants and HBeAg seroconversion has not been investigated in adult populations. Although a statistically significant association was not observed, our findings indicate that the minor T allele for rs3077 might increase the likelihood of early spontaneous HBeAg seroconversion in children. The sample size was estimated by the software G*Power 3.1.9.2, considering the effect size 0.3, the minimum power 0.80, and α = 0.05, resulting in 143. However, this study evaluated 109 patients. Greater statistical power might be needed to detect a significant association between *HLA-DP* genetic variants and early spontaneous HBeAg seroconversion.

*IL10* promoter and *IL28B* polymorphisms have been reported to be associated with HBV infection control [5, 6, 26]. Genetic variants of the *IL-10* gene promoter (*IL-10-819* and −*592*) were associated with disease progression in adult patients with chronic HBV infection [5]. In addition, genetic variants of the *IL-10* gene promoter (*IL-10-592*) were associated with a higher risk of persistent HBV infection in adults [6]. A pediatric study in Taiwan demonstrated that the *IL-10-1082* genotype GG and *IL-12-10993* genotype CG, which influence the serum levels of IL-10 and IL-12, were associated with early spontaneous HBeAg seroconversion [8]. However, this study failed to reveal a significant association between early HBeAg seroconversion and cytokine genetic variants.

Interactions among HLA-restricted T lymphocytes, antibody-secreting B lymphocytes, natural killer cells, and cytokines influence the immune response to HBV infection. The effective presentation of viral antigens to CD4+ T cells and CD8+ T cells by HLA class II and class I molecules, respectively, plays a crucial role in the immune response to HBV [1, 11]. Both *HLA-DP* and *HLA-DQ* encode class II molecules that have been implicated in the response to HBV infection. Although the associations of HLA alleles with the outcomes of HBV infection and responsiveness/non-responsiveness to the HB vaccine have been evaluated in numerous studies for two or three decades [27], information in children is limited [11]. The associations of HLA with vertical transmission (DRB1*03) [28], intrauterine transmission (DRB1*11) [29], and responsiveness to the vaccine (DRB1*01, DRB1*03, DRB1*11, DRB1*15, and DQB1*02) [30, 31] have been tested in infants and children in several studies. A previous study in Taiwan showed that HLA-B61 and HLA-DQB1*0503 are associated with early spontaneous HBeAg seroconversion in children with chronic HBV infection [32].

*HLA-DPA1* and -*DPB1* are less polymorphic than HLA-DR or –DQ, and the HLA-DP cell surface expression levels are likely to also be lower [33, 34]. Historically, this finding led to the notion that DP may have less clinical impact than another HLA. Therefore, the *HLA-DP* genes have not attracted a great deal of attention by researchers compared with *HLA-DR* and –*DQ*. Although hematopoietic stem cell transplantation [35–37], kidney transplantation [38, 39], juvenile idiopathic arthritis [40], and chronic beryllium disease [41, 42] have been reported to be related to HLA-DP after 2000, HLA-DP had never been investigated for its relationship with HBV disease before a GWAS study identified the close association between HLA-DP and HBV infection in Asians. Although the mechanism of the influence of the *HLA-DPA1* gene on HBV infection outcomes remains unclear, a recent study demonstrated that *HLA-DPA1* mRNA expression was found in normal liver and that the rs3077-G genotype decreased mRNA expression [43]. The decreased mRNA expression might be related to the risk of persistent HBV infection.

Of the 7 SNPs, 2 did not conform to HWE. No occurrence of mutations, no natural selection pressure, a large sample size, and random selection are required to test for HWE [44, 45]. However, this was a retrospective study, and the sample size was small. A majority of the children were referred to our hospitals from small hospitals or private clinics because of a failure to prevent mother-to-child transmission or the elevation of serum transaminases during follow-up. To collect samples for the G2 group, 37 HBV carrier mothers were enrolled as convenience samples in this study. Therefore, there was a significant difference in the sex ratio between the G1 and G2 groups. At the same time, 2 mother-child pairs and 3 sibling pairs were involved in this study. These conditions might contribute to the lack of HWE in the present study.

There are several limitations in this study. Multivariate analysis showed that there was a statistical significance in gender, duration of the follow-up period, serum HBV DNA levels, and transmission route between G1 and G2. Obviously, patient selection bias caused the significance difference in gender and duration of the follow-up period. Because it was impossible to examine serum HBV DNA levels at initial visit, serum HBV DNA levels at last visit were measured. Therefore, gender, duration of the follow-up period, and serum HBV DNA levels were not considered to be predictors for early HBeAg seroconversion. Although the source of HBV infection was unknown in 16 (28%) of 57 patients with late or no HBeAg seroconversion, multivariate analysis showed that mother-to-child transmission was a risk factor for late or no HBeAg seroconversion. This finding is consistent with previous studies, which reported that mother-to-child transmission mode is an important factors affecting HBeAg clearance rate in chronic HBV infection [46, 47]. In addition, pregnant women were also enrolled in G2, though the follow-up duration of pregnant mothers was shorter than children. As it was difficult to recruit HBeAg-seroconversion-negative patients in the pediatric department, to enroll a sufficient number of HBeAg-seroconversion-negative patients in this study, we recruited pregnant mothers. These factors might influence the results of this study.

## Conclusions

The *HLA-DPA1* SNP rs3077 showed a low p-value, but not significant, to have early spontaneous HBeAg seroconversion in children. This genetic variant might be a useful factor in the decision to treat children with chronic HBV infection.

## Abbreviations

HBV: Hepatitis B virus
HCC: Hepatocellular carcinoma
HLA: Human leukocyte antigen
IL28B: Interleukin 28 B
SNPs: Single-nucleotide polymorphisms.
